# Differences in antibiotic use between patients with and without a regular doctor in Hong Kong

**DOI:** 10.1186/s40360-015-0041-x

**Published:** 2015-12-15

**Authors:** Tai Pong Lam, Yuk Tsan Wun, Kwok Fai Lam, Kai Sing Sun

**Affiliations:** Department of Family Medicine and Primary Care, The University of Hong Kong, 3/F, Ap Lei Chau Clinic, 161 Main Street, Ap Lei Chau, Hong Kong; Department of Statistics and Actuarial Science, The University of Hong Kong, Pok Fu Lam, Hong Kong

**Keywords:** Antibiotic use, Continuity of care, General practice, Primary care physician

## Abstract

**Background:**

Literature shows that continuity of care from a primary care physician is associated with better patient satisfaction and preventive care. This may also have an effect on patients’ use of antibiotics. This study investigated the differences in antibiotic use between patients with and without a regular doctor in a pluralistic health care system.

**Methods:**

A cross-sectional telephone questionnaire survey using randomly selected household phone numbers was conducted in Hong Kong. Several key areas about antibiotic use were compared between the respondents with a regular doctor and those without.

**Results:**

The response rate was 68.3 %. Of the 2,471 respondents, 1,450 (58.7 %) had a regular doctor, 942 (38.1 %) without, and 79 (3.2 %) did not give a clear answer. The respondents with a regular doctor were more likely to report that they always finished the full course of antibiotics (74.2 % *vs* 62.4 %), as well as using antibiotics for their last upper respiratory tract infections (17.4 % *vs* 10.1 %). The association with antibiotic use remained significant in the multivariable logistic regression analysis after adjusting for other confounding factors (*P* < 0.001, OR = 1.76, 95 % CI:(1.27, 2.48)).

**Conclusions:**

While patients with a regular doctor, compared to those without, were more likely to report finishing the full course of antibiotics, they also had nearly twice the chance of reporting antibiotic use for upper respiratory tract infections. This challenges the common belief of the benefits in having a regular doctor.

**Electronic supplementary material:**

The online version of this article (doi:10.1186/s40360-015-0041-x) contains supplementary material, which is available to authorized users.

## Background

Many studies have shown that some patients expect antibiotic prescriptions for their upper respiratory tract infections (URTIs) and doctors often over-perceive their patients’ expectation of the drug [1, 2]. Some doctors would prescribe antibiotics based on non-biomedical reasons to satisfy the patients [3]. An unstable doctor-patient relationship [4] and the concerns that the unsatisfied patients would go elsewhere for future care might be the causes [1, 3, 5]. When a stable doctor-patient relationship has been established, the primary care physician could have a better chance to change the patient’s behaviours on antibiotics [6].

Previous studies showed that continuity of care in primary care was associated with increased patient satisfaction [7]. The concept of continuity of care emphasizes the presence of a regular doctor who the patient sees most of the time [8]. Patients who attended a regular primary care physician were more likely to receive better preventive care including cervical and breast cancer screening, vaccination, blood pressure measurement, cholesterol level checking, patient education on diet and exercise. Besides, they had fewer visits to emergency department [9–11]. In some studies, patients attending urgent-care or walk-in clinics were more likely to receive antibiotics for URTIs than those attending their routine primary care physicians [12–14].

The increasing threat of antibiotic resistance draws global concern. Misuse of antibiotics in primary care is no doubt a major area to be acted upon since more antibiotics are used in the community than in hospital settings. Campaigns that aimed to reduce improper antibiotic use and improve patient adherence to treatment [15–18] had done little to find out the possible impact of having a regular doctor. Our study was designed to collect evidence on the differences in antibiotic use between patients with and without a regular doctor in a pluralistic health care system, which is the major form of system in many parts of the world including the US and most Asian countries [19].

The general public in Hong Kong can consult any doctor in the private sector at their own choice, and over half of them have a regular doctor [19]. Similar to the US practice, a primary care physician in Hong Kong could be a family physician, a general practitioner, a general paediatrician, a general internal medicine physician, or a specialist of any field who also renders primary care to his/her patients. About 75 % of primary care service in Hong Kong is provided by the private sector (solo practitioners, group practices similar to health maintenance organizations, or general outpatient clinics in private hospitals), and the rest by the government general outpatient clinics at minimal charges. The private primary care physicians are paid on a fee-for-service basis while the public doctors earn a monthly salary based on their ranks.

This study was part of a larger project investigating the public’s knowledge, attitudes and practice related to antibiotic use in Hong Kong [20, 21]. Several key areas about antibiotic use were compared between two groups of patients, one with and the other without a regular doctor. We hypothesized that patients with a regular doctor would 1) use less antibiotics for URTIs, 2) have better adherence in finishing a full course of antibiotics for any illness, and 3) have stronger intention to prevent antibiotic resistance.

## Methods

### Study design and participants

A questionnaire for a territory-wide telephone survey in Hong Kong was designed based on the literature and our published findings from in-depth focus group discussions conducted at an earlier stage [20, 21]. The questionnaire was appraised by academic tutors in family medicine for its face- and content-validity before pilot-testing with 50 randomly selected household telephone respondents; revisions were made after each step. The responses to the items in the questionnaire were mainly in the form of yes (agree), no (disagree), uncertain or refused to answer. The determinants for grouping the respondents into those who had a regular doctor and those without included the following two questions: 1) Do you have a doctor you see most of the time?; 2) Do you usually see western doctor or traditional Chinese medicine practitioner? The first question asked about consistency and continuity in order to determine if the respondent was being taken care of by the same physician, i.e. a regular doctor. This question was probably most easily understood by the public in a telephone survey [19, 22]. For analytical purpose, the second question was used to restrict our definition of regular doctor to those who practiced western medicine. Traditional Chinese medicine practitioners are not authorised to prescribe antibiotics in Hong Kong. Although most Hong Kong citizens would consult western doctors for their illnesses, there are also about 15 - 20 % who would consult Chinese medicine practitioners for complementary treatment [23]. The relevant questions on antibiotic use and attitudes towards antibiotic resistance are shown in an additional file [see Additional file 1].

Random phone calls to households were conducted by the Social Sciences Research Centre of The University of Hong Kong, during the evenings of November and December, 2010. All the interviewers were trained and had practices before the survey. With the help of computer software, phone numbers were randomly selected from the latest residential telephone directory with a local service penetration over 98 %. The inclusion criteria for the survey were residents speaking Cantonese, the local dialect, and aged 18 years or above, while those who had not heard of antibiotics or with communication difficulties were excluded. The household member who fulfilled the above criteria and with a birthday coming soonest (“next birthday” method) was invited to take part in the survey. A maximum of five calls were made for any unanswered phone numbers. Ethics approval of this study was obtained from the Institutional Review Board of The University of Hong Kong/Hospital Authority Hong Kong West Cluster (Ref No. UW 07–359). Verbal informed consent was obtained from the respondents to participate in the telephone survey.

### Statistical analysis

The outcomes of the questionnaire survey were in terms of some proportions of the general population. To ensure that the estimation error of the proportion to be at most 0.02 with 95 % confidence, a sample size of 2,401 was required by assuming the most conservative “half-half” proportion from the respondents. The Pearson Chi-squared test was used to determine whether the nominal responses were associated with the status of having a regular doctor. Multivariable logistic regression analysis was used to estimate the effects of having a regular doctor by appropriately adjusting for the effects of other factors - age, sex, education, income and type of healthcare system attended (private *vs* public). A *P*-value <0.05 was taken as statistically significant.

For items which the respondents were uncertain or refused to answer, those responses were treated as missing values. In our analysis, respondents were grouped into three age-groups (<40, 40–65, >65 years old) and three monthly income-groups, i.e. low (<HK$10000 (US$1282)), middle (HK$10000-24999 (US$1282 - 3205)), and high (≥HK$25000 (US$3205)).

## Results

### Participants recruited

Of the 3,996 successful phone calls made to households, 376 of them did not meet the inclusion criteria. Among the remaining 3,620 calls, 813 respondents refused to take part and 336 did not complete the questionnaire, leaving 2,471 completed interviews (response rate 68.3 %) for analysis. The age distribution of the respondents was comparable to the Hong Kong population in the 2010 Census.

Out of the 2,471 respondents, 1,450 (58.7 %) had a regular western doctor, 942 (38.1 %) were without, and 79 (3.2 %) gave uncertain responses to that question. The respondents with a regular doctor were significantly more likely to have better education attainment, higher income, and utilise private healthcare services relative to those without a regular doctor (Table 1). In the multivariable logistic regression with age, sex, education, income and type of healthcare system, sex and education were not significant factors for having a regular doctor (*P* = 0.17 and *P* = 0.71 respectively). Respondents who were older (OR ratio for the young, middle and old age groups was estimated to be 1.00:1.26:3.14; *P* < 0.001), with higher income (OR ratio for low, middle and high income groups was 1.00:1.50:2.08; *P* < 0.0001) and attending private healthcare system (OR ratio for private and public system groups was 1.00:0.18, *P* < 0.0001) were more likely to have a regular doctor.

**Table 1:** Characteristics of questionnaire respondents with or without a regular western doctor

	^a^With a regular doctor	Without a regular doctor	*χ* ^2^ test	Adjusted Odds ratio^b^ (95 % CI)	Multivariable logistic regression *p*-value
Age			*χ* ^2^ = 0.213, P = 0.899		<0.001
<40 year	426 (31.6 %)	283 (32.2 %)		1.00	
40–64 year	813 (59.8 %)	517 (58.9 %)		1.26 (0.99,1.60)	
≥65 year	120 (8.8 %)	78 (8.9 %)		3.14 (1.92,5.20)	
Sex			*χ* ^2^ = 0.897, *P* = 0.344		0.17
Male	499 (34.4 %)	342 (36.3 %)		1.00	
Female	951 (65.6 %)	600 (63.7 %)		1.17 (0.93,1.46)	
Education			*χ* ^2^ = 10.461, *P* = 0.005		0.71
Primary or below	236 (16.5 %)	185 (19.9 %)		1.00	
Secondary	726 (50.7 %)	494 (53.1 %)		0.87 (0.61,1.22)	
Tertiary	469 (32.8 %)	251 (27.0 %)		0.89 (0.59,1.33)	
Income group			*χ* ^2^ = 46.710, P < 0.0001		<0.0001
<HK$10,000	188 (16.9 %)	197 (28.0 %)		1.00	
$10,000-24,999	398 (35.9 %)	275 (39.0 %)		1.50 (1.10,2.04)	
≥$25,000	523 (47.2 %)	232 (33.0 %)		2.08 (1.49,2.90)	
Healthcare system attended			*χ* ^2^ = 203.82, *P* < 0.0001		<0.0001
Private	1248 (88.6 %)	570 (63.8 %)		1.00	
Public	160 (11.4 %)	324 (36.2 %)		0.18 (0.13,0.23)	

^a^Some data in the categories were missing due to respondents’ refusal to answer

^b^The odds ratio here is defined as the ratio of the odds of having a regular doctor among those with a regular doctor to those without adjusted for age, sex, education, income group and healthcare system

### Practices

Table 2 shows the comparison of the practices between the two groups. The respondents with a regular doctor were significantly more likely to always finish the full course of antibiotics (74.2 % *vs* 62.4 %, *P* < 0.0001). The difference remained significant after controlling for other factors (age, sex, education, income and private/public system attended) by multivariable logistic regression (*P* < 0.0001, OR = 1.81, 95 % CI:(1.42, 2.29)). Besides, among the respondents who had not asked for antibiotics during consultations, those with a regular doctor were more likely to attribute their not asking to their trust in the doctor (94.7 % *vs* 92.6 %, *P* = 0.039), and marginal significance was also achieved in the multivariable logistic regression (*P* = 0.047, OR = 1.59, 95 % CI:(1.01, 2.49)).

**Table 2:** Antibiotic use by questionnaire respondents

	With a regular doctor	Without a regular doctor	*χ* ^2^ -test	Adjusted Odds ratio (95 % CI)^b^	Multivariable logistic regression *p*-value
Ever asked for antibiotics			*χ* ^2^ = 0.025, *P* = 0.619	1.17 (0.81, 1.70)	0.399
Yes	129 (8.9 %)	78 (8.3 %)			
No	1317 (91.1 %)	862 (91.7 %)			
Ever bought antibiotics over counter			*χ* ^2^ = 0.423, *P* = 0.516	0.98 (0.67, 1.45)	0.913
Yes	108 (7.5 %)	77 (8.2 %)			
No	1336 (92.5 %)	861 (91.8 %)			
Did not ask but expected antibiotics^a^			*χ* ^2^ = 1.700, *P* = 0.192	1.15 (0.88, 1.50)	0.316
Yes	293 (22.9 %)	167 (20.4 %)			
No	989 (77.1 %)	650 (79.6 %)			
Did not ask for antibiotics because trusting the doctor^a^			*χ* ^2^ = 4.267, *P* = 0.039	1.59 (1.01, 2.49)	0.047
Yes	1235 (94.7 %)	765 (92.6 %)			
No	69 (5.3 %)	62 (7.4 %)			
Used antibiotics for the last URTI			*χ* ^2^ = 22.715, *P* < 0.000	1.76 (1.27, 2.48)	<0.001
Yes	231 (17.4 %)	89 (10.1 %)			
No	1093 (82.6 %)	789 (89.9 %)			
Always finished the full course			*χ* ^2^ = 32.723, *P* < 0.000	1.81 (1.42, 2.29)	<0.0001
Yes	993 (74.2 %)	502 (62.4 %)			
No	346 (25.8 %)	302 (37.6 %)			
Finished the full course because told by clinical staff to do so			*χ* ^2^ = 7.078 *P* = 0.008	1.45 (0.95, 2.19)	0.086
Told to finish	888 (89.7 %)	424 (85.0 %)			
Not told to finish	102 (10.3 %)	75 (15.0 %)			

^a^Respondents who had asked for antibiotics did not answer these questions

^b^Odds ratio adjusted for age, sex, education, income group and healthcare system

However, there was no significant difference in requesting and expecting antibiotics from doctors as well as acquiring over-the-counter antibiotics between the respondents with and without a regular doctor. Interestingly, the respondents with a regular doctor were much more likely to report using antibiotics in their last consultation for URTI (17.4 % vs 10.1 %, *P* < 0.0001). The association remained significant in the multivariable logistic regression analysis after adjusting for other factors (*P* < 0.001, OR = 1.76, 95 % CI:(1.27, 2.48)).

### Attitudes

There were some differences between respondents with and without a regular doctor in their attitudes towards antibiotic resistance (Table 3). Respondents with a regular doctor were more likely to prefer doctors who rarely prescribed antibiotics (38.2 % *vs* 34.5 %, *P* = 0.033), agree that less prescription by doctors lowered the probability of antibiotic resistance (86.9 % *vs* 82.9 %, *P* = 0.018), and feel that they could help the prevention of antibiotic resistance (40.4 % *vs* 35.7 %, *P* = 0.043). However, multivariable logistic regression showed that there was no significant association between these responses and the factor of having a regular doctor after the adjustment for other factors.

**Table 3:** Attitudes towards antibiotic resistance

	With a regular doctor	Without a regular doctor	*χ* ^2^ -test	Adjusted Odds ratio (95 % CI)^b^	Multivariable logistic regression *p*-value
Preference for doctors who			*χ* ^2^ = 10.503, *P* = 0.033	NA	0.10
Rarely prescribe antibiotics	552 (38.2 %)	323 (34.5 %)			
Readily prescribe antibiotics	29 (2.0 %)	17 (1.8 %)			
Prescribe antibiotics on request	59 (4.1 %)	58 (6.2 %)			
Indifferent to such choice	695 (48.2 %)	449 (47.9 %)			
Uncertain about such choice	108 (7.5 %)	90 (9.6 %)			
Taking fewer courses of antibiotics helps reduce antibiotic resistance^a^			*χ* ^2^ = 0.032, *P* = 0.858	0.87 (0.59, 1.25)	0.445
Yes	1055 (88.6 %)	669 (88.8 %)			
No	136 (11.4 %)	84 (11.2 %)			
Less prescription by doctors lowers the probability of antibiotic resistance^a^			*χ* ^2^ = 5.624, *P* = 0.018	1.37 (0.99, 1.88)	0.062
Yes	1011 (86.9 %)	599 (82.9 %)			
No	152 (13.1 %)	123 (17.1 %)			
You can help the prevention of antibiotic resistance^a^			*χ* ^2^ = 4.101, *P* = 0.043	1.04 (0.82, 1.33)	0.727
Yes	455 (40.4 %)	251 (35.7 %)			
No	670 (59.6 %)	452 (64.3 %)			

^a^Respondents who had no idea about the meaning of “antibiotic resistance” did not answer these questions

^b^Odds ratio adjusted for age, sex, education, income group and healthcare system

## Discussion

Our study indicated that there were both pros and cons in antibiotic use by having a regular doctor. After controlling for the confounding factors of age, sex, education, income and private or public system attended, the respondents with a regular doctor only exhibited more positive results than other respondents in one aspect: more likely to report finishing the full course of antibiotics, which matched with our hypothesis 2. However, contrary to hypothesis 1, the respondents with a regular doctor were much more likely (OR = 1.76, 95 % CI:(1.27, 2.48)) to report using antibiotics in their last consultation for URTI. This finding did not echo the common belief that an established doctor-patient relationship in primary care would minimize inappropriate use of antibiotics [6, 13]. For hypothesis 3 that patients with a regular doctor had stronger intention to prevent antibiotic resistance, it was refuted after adjusting for confounding factors.

It is known that non-biomedical reasons can affect doctors’ antibiotic prescriptions for patients [3]. This study focused on the potential effect of having a regular doctor. Although we strongly acknowledge the benefits of continuity of care which enhances patient satisfaction and preventive care, [7, 9–11] our findings alert us to its potential drawbacks which might be neglected or under-reported in previous studies [24]. A recent qualitative study reported that primary care physicians felt easier to say “No” to long standing patients than to new patients when requests for antibiotics were made [6]. Accordingly, patients with regular doctors should be more likely to be refused when making improper request for antibiotics. However, the quantitative finding of this study showed the opposite, and this was consistent with our previous survey which found that patients who were attending doctors for follow-up consultations for infective illnesses were more likely to be prescribed antibiotics [25]. A possible explanation might be a concern that the unsatisfied patients would go elsewhere for future care, [1, 3, 5] which would have significant financial implications for the primary care physicians in a pluralistic healthcare system. Although we do not have clear evidence for the causes, our findings suggested a possible negative impact from the higher antibiotic prescription rate for URTI by regular doctors and it should be further studied in other countries. This may contribute to the global mission of reducing unnecessary antibiotic prescription in the primary care setting.

The proportion of respondents reporting use of antibiotics for URTIs in this study (17.0 %, Table 2) was slightly lower than the 23.7 % found in another local telephone survey conducted among the general public in 2008 [26]. Doctors in Hong Kong were getting increasingly cautious with the use of antibiotics [25]. In fact, among the respondents in the current survey who claimed that they did not ask for antibiotics during consultations, over 92 % of them considered “trust in the doctor” as the reason for not asking. In addition, a greater proportion of these respondents had regular doctors though the difference was of marginal statistical significance (OR = 1.59, 95 % CI:(1.01, 2.49)). Therefore, the doctors should value the patients’ trust and avoid over-perceiving their demand for antibiotics [1, 2].

### Limitations of the study

Firstly, it is difficult to define without controversy what a “regular doctor” means. We used an intuitive definition in our questionnaire, “the doctor [whom] you see most of the time” to mean personal continuous care by a primary care physician. This definition is admittedly loose but probably most easily understood by the public in a telephone survey [19, 22]. A regular doctor was presumed to deliver continuous care instead of repeated episodic care. It is possible that patients having a regular doctor would still see other doctors on occasions (e.g. seeing the regular doctor for 80 % of the time but went elsewhere for the remaining 20 %).

Secondly, the study findings were based on self-reported data from the respondents. The findings were not validated with triangulation using actual clinical data. However, the potential recall bias should be small as the questions were asking about their usual practice or most recent experiences.

Thirdly, we assumed antibiotics were not needed for almost all cases of common cold/flu. Medical evidence suggests that antibiotics are not indicated for URTI except in the very rare cases like compromised immunity or for the very sick patients with serious comorbid diseases. Most URTIs among adults in the community are self-limited viral infections that can be treated without antibiotics [27]. The respondents of this study were from the ambient general population, hence the possibility of a medical indication for antibiotics for a URTI would be very rare.

Fourthly, this study focused on the impact of having a regular doctor. We used the multivariable logistic regression to adjust the effects of age, sex, education, income and type of healthcare system attended (private *vs* public). Nonetheless, we did not ask about the medical history of the respondents in the telephone survey. There was a possibility that the health status, URTI consultation rate of the respondents with a regular doctor were different from those without. If marked disparities did exist, the differences in antibiotic use observed between the two groups might not be due to having a regular doctor. It is worthwhile to conduct further studies in this respect. Finally, our findings in Hong Kong might not be generalizable to other countries.

## Conclusions

This study showed that while Hong Kong patients with a regular doctor were more likely to report finishing the full course of antibiotics, they also had nearly twice the chance of reporting using antibiotics for URTI. This finding is contrary to the common belief in the benefits of having a regular primary care physician and should be further researched in other countries.

## Additional file

Additional file 1:
**Question items on antibiotic use and attitudes towards antibiotic resistance.** (PDF 95 kb)

## Abbreviation

URTI: Upper respiratory tract infection
