# Supplementary material for: Differences in antibiotic use between patients with and without a regular doctor in Hong Kong
Source: BMC Pharmacol Toxicol. 2015 Dec 15;16:40. doi: 10.1186/s40360-015-0041-x (PMC4681134; doi:10.1186/s40360-015-0041-x)
Supplement: Additional file 1: — Question items on antibiotic use and attitudes towards antibiotic resistance. (PDF 95 kb) [file 40360_2015_41_MOESM1_ESM.pdf]

<u>Question items on antibiotic use and attitudes towards antibiotic resistance</u>

Antibiotic use

1. Have you ever asked the doctor to prescribe antibiotics for yourself?
   yes
   no *[→ask follow-up questions]*
   don't know / difficult to say
   refused to answer

   →1.1 Did you expect antibiotics but had not asked for it?
   yes
   no
   don't know / difficult to say
   refused to answer

   →1.2 You did not ask for antibiotics because trusting the doctor?
   yes
   no
   don't know / difficult to say
   refused to answer

2. Have you ever bought antibiotics over the counter?
   yes
   no
   don't know / difficult to say
   refused to answer

3. Last time you had a “common cold”, did you use antibiotics?
   yes
   no
   don't know / difficult to say
   refused to answer

4. When you were prescribed antibiotics in the past, how often did you finish the full course on time?
   every time *[→ask follow-up question]*
   most of the time
   sometimes
   never
   doctors have never prescribed antibiotics to me
   don’t know / difficult to say
   refused to answer

   →4.1 Did you finish the full course because told by clinical staff to do so?
   yes
   no
   don't know / difficult to say
   refused to answer

Attitudes towards antibiotic resistance

1. Regarding antibiotics use, what kind of doctor do you prefer?
   rarely prescribe antibiotics
   readily prescribe antibiotics
   prescribe antibiotics upon patient’s request
   it is indifferent whether the doctor prescribes antibiotics or not
   don’t know / difficult to say
   refused to answer

2. Regarding the usage of antibiotics, do you agree with following sentences?

   2.1 Taking fewer courses of antibiotics would help reduce antibiotic resistance.
      agree
      disagree
      don’t know what is meant by antibiotic resistance *[skip the following two questions]*
      don’t know / difficult to say
      refused to answer

   2.2 The lesser antibiotics prescription by doctors, the lower probability of antibiotic resistance in the community.
      agree
      disagree
      don’t know / difficult to say
      refused to answer

   2.3 You can help the prevention of antibiotic resistance in the community.
      yes
      no
      don't know / difficult to say
      refused to answer
